# Prokineticin Receptor Inhibition With PC1 Protects Mouse Primary Sensory Neurons From Neurotoxic Effects of Chemotherapeutic Drugs *in vitro*

**DOI:** 10.3389/fimmu.2020.02119

**Published:** 2020-09-24

**Authors:** Giorgia Moschetti, Theodora Kalpachidou, Giada Amodeo, Roberta Lattanzi, Paola Sacerdote, Michaela Kress, Silvia Franchi

**Affiliations:** ^1^Department of Pharmacological and Biomolecular Sciences, Università Degli Studi di Milano, Milan, Italy; ^2^Department of Physiology and Biomedical Physics, Medical University of Innsbruck, Innsbruck, Austria; ^3^Department of Physiology and Pharmacology “Vittorio Erspamer”, Sapienza University of Rome, Rome, Italy

**Keywords:** chemotherapy, DRG, neurons, prokineticins, neurotoxicity

## Abstract

Neurotoxicity is a common side effect of chemotherapeutics that often leads to the development of chemotherapy-induced peripheral neuropathy (CIPN). The peptide Prokineticin 2 (PK2) has a key role in experimental models of CIPN and can be considered an insult-inducible endangering mediator. Since primary afferent sensory neurons are highly sensitive to anticancer drugs, giving rise to dysesthesias, the aim of our study was to evaluate the alterations induced by vincristine (VCR) and bortezomib (BTZ) exposure in sensory neuron cultures and the possible preventive effect of blocking PK2 signaling. Both VCR and BTZ induced a concentration-dependent reduction of total neurite length that was prevented by the PK receptor antagonist PC1. Antagonizing the PK system also reduced the upregulation of PK2, PK-R1, TLR4, IL-6, and IL-10 expression induced by chemotherapeutic drugs. In conclusion, inhibition of PK signaling with PC1 prevented the neurotoxic effects of chemotherapeutics, suggesting a promising strategy for neuroprotective therapies against the sensory neuron damage induced by exposure to these drugs.

## Introduction

Neurotoxicity is a common side effect of chemotherapeutic agents generally used in clinic for the treatment of cancer (1). Such neurotoxicity often leads to the development of chemotherapy-induced peripheral neuropathy (CIPN), which is one of the most common and debilitating dose-limiting side effects of first-line anticancer drugs (2–5). CIPN represents a complex syndrome characterized by many sensory abnormalities (6, 7), which make life difficult for patients that are already experiencing cancer. Neuropathy development has been associated with vinca alkaloids [e.g., vincristine (VCR)], proteasome inhibitors [e.g., bortezomib (BTZ)], platinum agents (e.g., oxaliplatin), and taxanes (e.g., paclitaxel) (8–11). Despite having different anti-tumoral mechanisms of action, all these drugs induce neuropathy both in humans and in experimental rodent models. Experimental painful peripheral neuropathies are already produced by relatively low doses that do not cause axonal degeneration in peripheral nerve. Partial degeneration of the intraepidermal innervation suggests mechanisms that might produce chemotherapy-evoked neuropathic pain, and activation of cutaneous Langerhans cells suggests possible neuroimmune interactions that might also have a role (12). Peripheral sensory neuron terminals appear to be particularly sensitive to damage induced by antineoplastic agents because of the lack of an efficient neurovascular barrier and the presence of a dense network of fenestrated capillaries, which lead to an unrestrained drug permeation (3, 13, 14). Even if the molecular events underlying CIPN are still largely unknown, the involvement of mitochondrial toxicity, oxidative stress, ion channel alterations, and neuroinflammatory processes has been discussed (4, 13, 15). Mammalian Prokineticin 2 (PK2) is a secreted bioactive peptide that belongs to the prokineticin (PK) family (16, 17) and has been shown to be involved not only in the onset but also in the persistence of painful conditions of several origins (18–22). PK2 is a ligand of two closely related G-protein-coupled receptors (GPCRs) known as PK-R1 and PK-R2 (23, 24). Both PK2 and its receptors are expressed in neurons and accessory cells (satellite cells, microglia, and astrocytes) in the nervous system and immune cells, such as granulocytes, macrophages, and lymphocytes (16, 25). In primary sensory afferents, PK2 as well as PK-R1 and PK-R2 receptors are expressed in different neuron types: in dorsal root ganglia (DRG) harboring the cell bodies of primary afferent neurons, PK-R1 is mainly expressed on small-size nociceptors, while PK-R2 is expressed on medium- and large-sized neurons (25, 26). PK2 receptor may couple to Gq, Gi, and Gs (27, 28) depending on their cellular localization and thus can activate different intracellular signaling pathways (16, 25). For example, in sensory neurons, PK2 receptors are Gq-coupled and promote intracellular Ca^2+^ mobilization through the activation of phospholipase C (PLC) and formation of IP3. PK2 receptor activation, via Gq, induces protein kinase C (PKC)-ε translocation to the plasma membrane (29). PK2 sensitizes TRPV1- and TRPA1-expressing nociceptors (17, 29) and induces the release of pro-inflammatory/algogen mediators, like cytokines, substance P (SP), and calcitonin gene-related peptide (CGRP) (29). A dramatic increase of PK2 in sensory neurons, concurrent with the development of painful symptoms, was established in neuropathic conditions, such as peripheral nerve injury (19–21) and diabetic neuropathy (18). Moreover, we recently demonstrated the involvement of PK2 as well as therapeutic effect of its receptor antagonist PC1 in *in vivo* murine models of CIPN induced by BTZ (30) and VCR (31). Besides, a possible role for PK2 as an insult-inducible endangering mediator for cerebral ischemic injury has been proposed (32). PK2 and its receptors are associated with amyloid beta (Aβ) neurotoxicity and mRNA and protein levels of PK2 and its receptors were significantly modified by Aβ treatment (33, 34), suggesting that upregulation of PK2 could be common to numerous pathological conditions, such as hypoxia, reactive oxygen species, and excitotoxic glutamate (35).

Based on the involvement of PK2 in murine *in vivo* CIPN models and the possible role of this protein as an insult-inducible endangering mediator, the aim of this study was to investigate whether PK2 may be involved in direct neurotoxicity induced by two different cytostatic drugs: VCR (microtubules formation inhibitor) and BTZ (26s proteasome inhibitor) on sensory neurons. Given the well-established alterations of neuronal cells following chemotherapy exposure (36–45), we decided to use mouse primary sensory neuron cultures and evaluate the effects of PK-Rs antagonism on neurite outgrowth and immune mediator expression induced by VCR and BTZ.

## Materials and Methods

### Animals

Male 9-week-old C57BL/6J mice (Charles River Laboratories, Calco, Italy; Janvier Labs, France) were used in the experiments. Animals were housed in groups of three in translucent polyethylene cages. They were housed with light/dark cycles of 12 h, temperature of 22 ± 2° C, humidity of 55 ± 10%, and standard rodent chow and tap water *ad libitum*. Animals were acclimatized to the environment for 7 days before starting the experiments. All the procedures performed on animals were in compliance with international policies (EEC council directive 86/609, OJ L 358, 1 Dec. 12, 1987; Guide for the Care and Use of Laboratory Animals, Us National Research Council, 8th ed., 2011) and were approved by the Animal Care and Use Committee of the Italian Ministry of Health (permission number 709/2016) and by the Austrian National Animal Experiment Ethics Committee of the Austrian Bundesministerium für Wissenschaft und Forschung (permit number BMWF-66.011/0054-WF/V/3b/2015). All efforts were made to reduce the number of animals used and to minimize animal suffering in accordance with the 3R principles.

### Chemicals

VCR (Tocris Bioscience, Minneapolis USA) and BTZ (LC Laboratories, Woburn, USA) were freshly prepared before each experiment by dissolving them in sterile PBS solution. These stock solutions were then diluted in culture medium and added to the cell cultures 24 h after plating DRG primary sensory neurons. Control cells were incubated with vehicle (PBS sterile solution and culture medium). Different concentrations of VCR (0.01, 0.1, 1, 5, 10, 50, and 100 nM and 1, 5, 10, 20, 50, and 100 μM) and of BTZ (4, 6, 8, and 10 nM) were tested (39, 40). The PK-Rs antagonist PC1, a triazine-guanidine compound, was provided by G. Balboni, Università di Cagliari, Cagliari, Italy (46). PC1 is a triazine–guanidine derivative that *in vitro* blocks PK2-induced intracellular Ca^++^ increase in PK-R1- and PK-R2-transfected CHO cells and *in vivo* antagonizes hyperalgesia induced by PK2. Affinity studies for PK-Rs indicated a Ki of 22 and 1,610 nM for PK-R1 and PK-R2, respectively (30). The antagonist was dissolved in sterile deionized water, diluted in culture medium, and added to cell cultures. Different concentrations of PC1 (50, 100, 250, and 500 nM and 1 μM) were tested (34, 47). Control cells were incubated with vehicle containing sterile deionized water and culture medium only.

### DRG Cell Cultures

**Figure 1:**
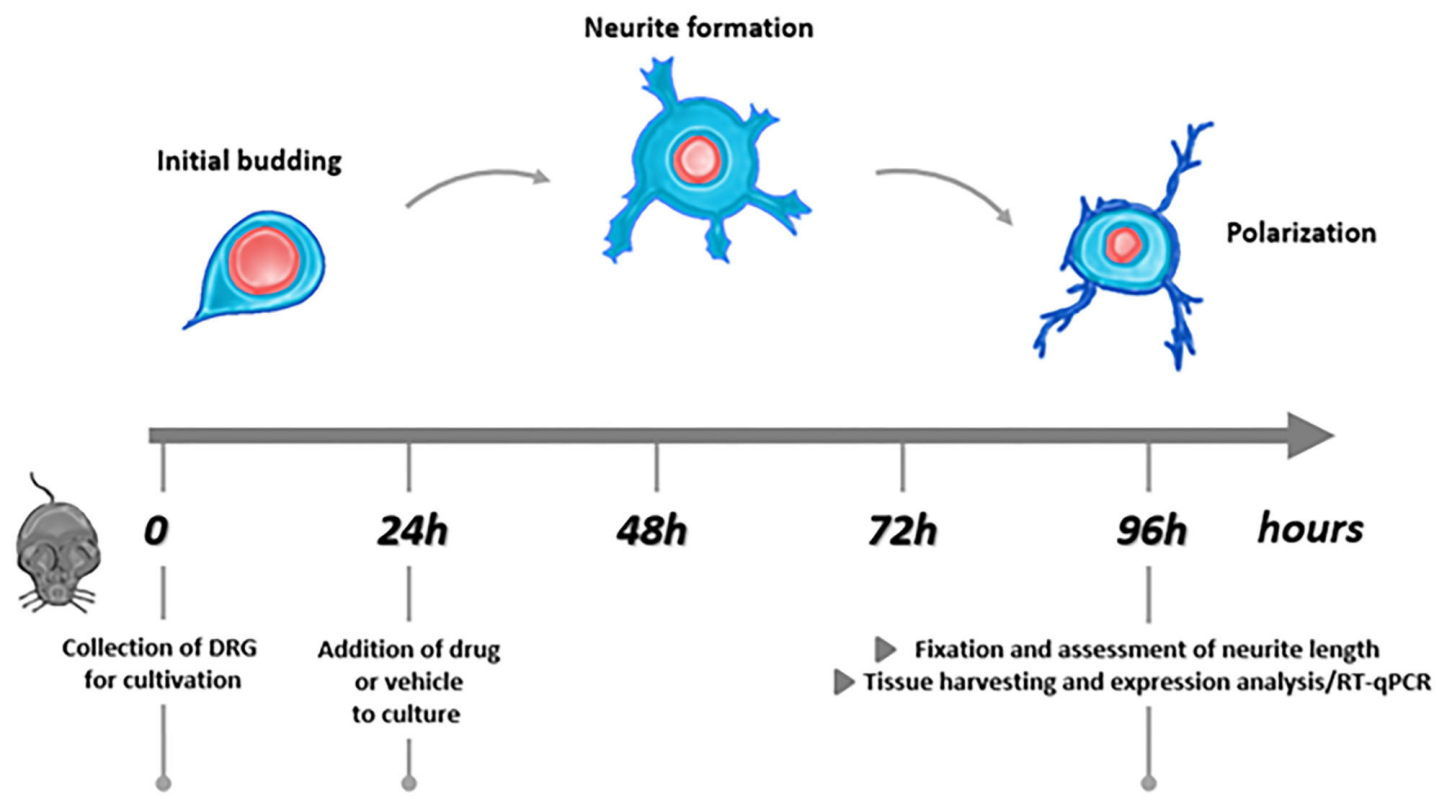
Experimental protocol.

Lumbar (L1–L6) DRG were collected from healthy mice (48) and processed as previously described (49, 50). Briefly, ganglia were cleaned from connective tissue and incubated in Liberase Blendzyme 1 (9 mg/100 ml DMEM, Roche) for 1 h, at 37° C, 5% CO_2_. After washing with PBS, Trypsin-EDTA (Invitrogen) was added and samples were incubated at 37° C, 5% CO_2_ for 15 min. The serum-free TNB 100 basal medium (Biochrom) containing L-glutamine (Invitrogen), penicillin G sodium, streptomycin sulfate (Invitrogen), and protein–lipid complex (Biochrom) was used for washing and cell culturing. The lipid–protein complex acts as a serum substitute that contains a chemically defined amount of important fatty acids, proteins, and vitamins. Inhibitors of non-neuronal cell division were not included in the medium. After mechanical dissociation using a fire-polished Pasteur pipette, the resulting cell suspension was centrifuged at 500 rpm through a 3.5% BSA gradient (Sigma-Aldrich) for 10 min. The pellet obtained was washed using TNB medium and centrifuged for 5 min at 760 rpm. The pellet was resuspended in TNB medium + murine NGF (25 ng/ml, Alomone Labs). Neurons were plated on 12-mm glass coverslips (for neurite outgrowth assay) or in 24-well plates (mRNA extraction, RT-qPCR), previously coated with 0.01% poly-L-lysine (Sigma-Aldrich) and 1:20 laminin 1 mg/ml (Sigma-Aldrich). Neurons were cultured in TNB medium with murine NGF (25 ng/ml, Alomone Labs) at 37° C in 5% CO_2_ for 24 h. After 24 h, cell health and the presence of first neurites were assessed using a light microscope and different doses of the chemotherapeutic drugs alone or in combination with PC1, PC1 alone, or the vehicle were added. Every 24 h, cells were controlled using a light microscope. After 96 h of incubation (i.e., 72 h of drugs exposure), cells were processed for neurite outgrowth assay or RNA extraction and RT-qPCR. A schematic representation of the experimental protocol is presented in Figure 1.

### Neurite Length and PK2 Evaluation by Immunofluorescence

DRG neurons were fixed in 4% PFA for 10 min at room temperature and, after washing with PBS, permeabilized using 0.1% Triton-X-100 (3 min incubation). After further washing, blocking buffer (1% BSA in PBS) was added for 30 min. For total neurite length assessment, cells were incubated with neuron-specific β-III tubulin (TuiJ-1) monoclonal mouse antibody (1:1,000, R&D Systems) for 1 h at room temperature. A chicken secondary antibody anti-mouse IgG Alexa Fluor™ 594 (1:1,000, Thermo Fisher Scientific) was used. Nuclei were counterstained with DAPI (1 μg/ml, Tocris Bioscience), and finally coverslips were mounted using Mowiol 40–88 (Sigma-Aldrich). Digitalized images of randomly chosen areas of the coverslip were recorded using an Axio Imager microscope (Carl Zeiss) with 20 × objective and a CCD camera (SPOT; Diagnostic Instruments). Only neurons that were clearly separated from neighboring cells were included in the analysis; neurons with no visible process or with only filopodial formations were counted as negative (49, 50). Quantification was performed using ImageJ software and NeuronJ plugin [http://www.imagescience.org/meijering/software/neuronj/;(51)]. The neurite length from at least 80 single neurons for each *in vitro* treatment condition was measured. Four independent experiments were performed. Total neurite length was calculated for each cell, and then neurite length of the cells belonging to the same experimental conditions were averaged and these values are expressed as mean ± SEM of all the cells evaluated with *n* ≥ 80. This procedure has been used in several other published papers from ours and other groups (49, 50). For PK2 localization, cells were in addition colabeled with a primary rabbit polyclonal antibody for PK2 (1:400, Abcam) overnight at 4° C. A donkey anti-rabbit IgG Alexa Fluor™ 488 secondary antibody (1:400, Thermo Fisher Scientific) was used. Labeling control (sample section incubated in all of the buffers and detergents used in the experiment, but not in secondary antibody) were performed before starting the experiments. The secondary antibody control (primary antibody replaced with the same amount of 1% BSA) was included in each experiment.

### RNA Extraction and Quantification

For end point analysis of mRNA expression, total RNA was isolated from cell cultures using TRIzol® reagent (Invitrogen, Thermo Fisher Scientific, Italy) according to the manufacturer's instructions and re-suspended in 20 μl of RNase-free water. RNA samples underwent DNAse treatment (DNA-free™ DNase kit Treatment and Removal Reagents, Ambion, Applied Biosystem, Italy) to avoid false-positive results due to contaminating DNA genomic amplification. RNA quantity and quality were determined using a BioPhotometer (Eppendorf, Germany). From each sample, an equal amount of RNA (1,000 ng) underwent reverse transcription using iScript™ Reverse Transcription Supermix for RT-qPCR (Bio-Rad, Italy), and the cDNA was used as template for the reverse transcription quantitative polymerase chain reaction (RT-qPCR). Genes of interest were analyzed by RT-qPCR using the following TaqMan Gene Expression Assays (Thermo Fisher Scientific, Waltham, USA): PK2 (Prok2, Mm01182450_g1), PK-R1 (Prokr1, Mm00517546_m1), PK-R2 (Prokr2, Mm00769571_m1), IL-1β (Mm00434228_m1), IL-6 (Mm00446190_m1),TNF-α (Mm00443258_m1), IL-10 (Mm00439616:m1), TLR4 (Mm00445274_m1), GFAP (Mm01253033_m1), ATF3 (Mm00476033_m1), and GAPDH (Mm99999915_g1). Amplification was performed using ABI PRISM 7000 Sequence Detection System (Applied Biosystem, Foster City, CA) and carried out in a final volume of 20 μl consisting of 2 μl of cDNA (corresponding to 100 ng of cDNA), 10 μl of Luna®Universal Probe qPCR Master Mix (New England BioLabs, Ipswich, MA), 1 μl of TaqMan Gene Expression Assays (Thermo Fisher Scientific), and RNAse-free water. Experimental procedures were performed according to the TaqMan Gene Expression Assays protocol. Each sample was run in triplicate alongside non-template controls. The PCR cycle protocol used was as follows: 1 min at 95° C, 40 five-step cycles of 15 s at 95° C, and 30 s at 60° C. Threshold cycle numbers (Ct of the specific gene of interest and the endogenous control gene GAPDH) were determined by ABI PRISM 7000 Sequence Detection System (Applied Biosystems®, Foster City, USA). The Ct value of the specific gene of interest was normalized to the Ct value of the endogenous control, GAPDH, and the comparative Ct method (2^−ΔΔCt^) was then applied using the specific control group (vehicle treated cells) as a calibrator.

### Data Analysis

Statistical analysis was performed using GraphPad Prism 6 Software (San Diego, CA, USA). Data are presented as mean ± SEM. For the neurite outgrowth assay, at least 80 cells (*n* ≥ 80), obtained from four independent experiments, were scored per experimental condition. Total neurite length was calculated for each cell, and then the neurite lengths of the cells belonging to the same experimental conditions were averaged, and these values are expressed as mean ± SEM of all the cells evaluated. For the RT-qPCR, eight cultures obtained from eight mice were used. Results were analyzed by using one-way analysis of variance (ANOVA), followed by Bonferroni's *post hoc* test for multiple comparison. The overall significance level was *p* < 0.05 for each hypothesis.

## Results

### Dose-Dependent Inhibition of Neurite Length in VCR Treated Neurons

**Figure 2:**
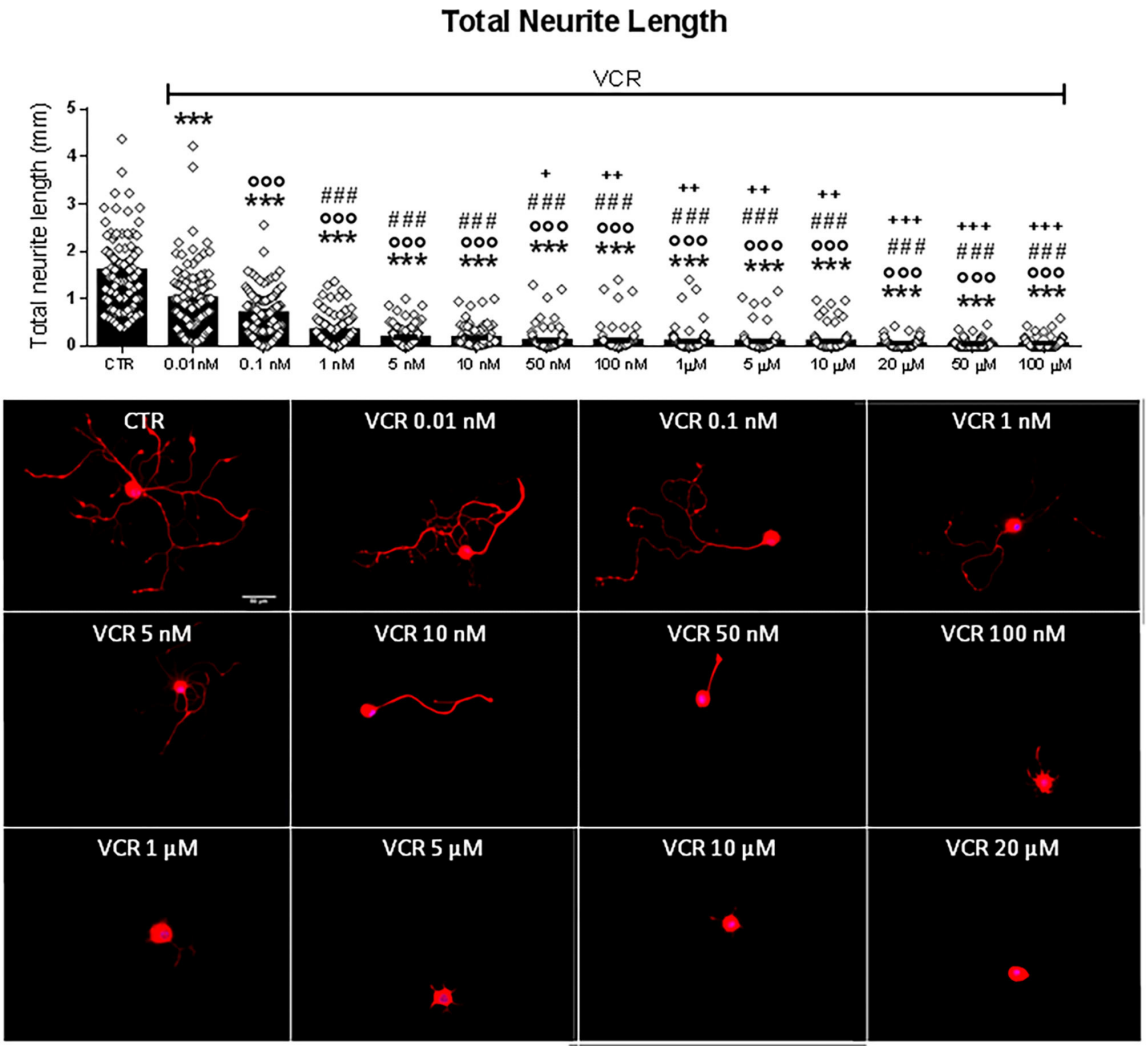
Quantification of total neurite length of DRG primary sensory neurons treated *in vitro* with the vehicle (CTR) or with the following VCR concentrations: 0.01, 0.1, 1, 5, 10, 50, and 100 nM and 1, 5, 10, 20, 50, and 100 μM. In the same figure are reported representative images of β3-tubulin (red) staining in DRG cell cultures. Cell nuclei were counterstained with DAPI (blue). Scale bar = 50 μm. Data are presented as mean ± SEM, *n* ≥ 80 cells obtained from four independent experiments. One-way ANOVA followed by Bonferroni's post-test. ***p < 0.001 vs. CTR; ^°°°^*p* < 0.001 vs. VCR 0.01 nM; ###*p* < 0.001 vs. VCR 0.1 nM; +*p* < 0.05, ++*p* < 0.01, +++*p* < 0.001 vs. VCR 1 nM.

The cytostatic drug VCR has been associated with clinical neuropathy and loss of epidermal innervation (12, 52). As a model for the sensitive peripheral nerve terminal, we used primary cultures of neurons obtained from adult murine DRG. Total neurite length was measured in cell cultures exposed to relevant doses of VCR. Figure 2 shows the quantification of total neurite length with representative examples of cells exposed to rising concentrations (from 0.01 nM to 100 μM) of VCR. As shown, VCR had toxic effects on neurons in a concentration-dependent manner; cells exposed to the lower tested doses 0.01 and 0.1 nM already showed a significant decrease in the total neurite length compared to controls [*F*_(13, 1, 272)_ = 145.4, *p* < 0.001; CTR vs. VCR 0.01 nM and vs. VCR 0.1 nM, *p* < 0.001]. With higher VCR concentrations, a further reduction of neuronal processes developed and neurites were virtually absent at VCR 50 nM or higher.

### Activation of the PK System in Neuron Cultures by VCR

**Figure 3:**
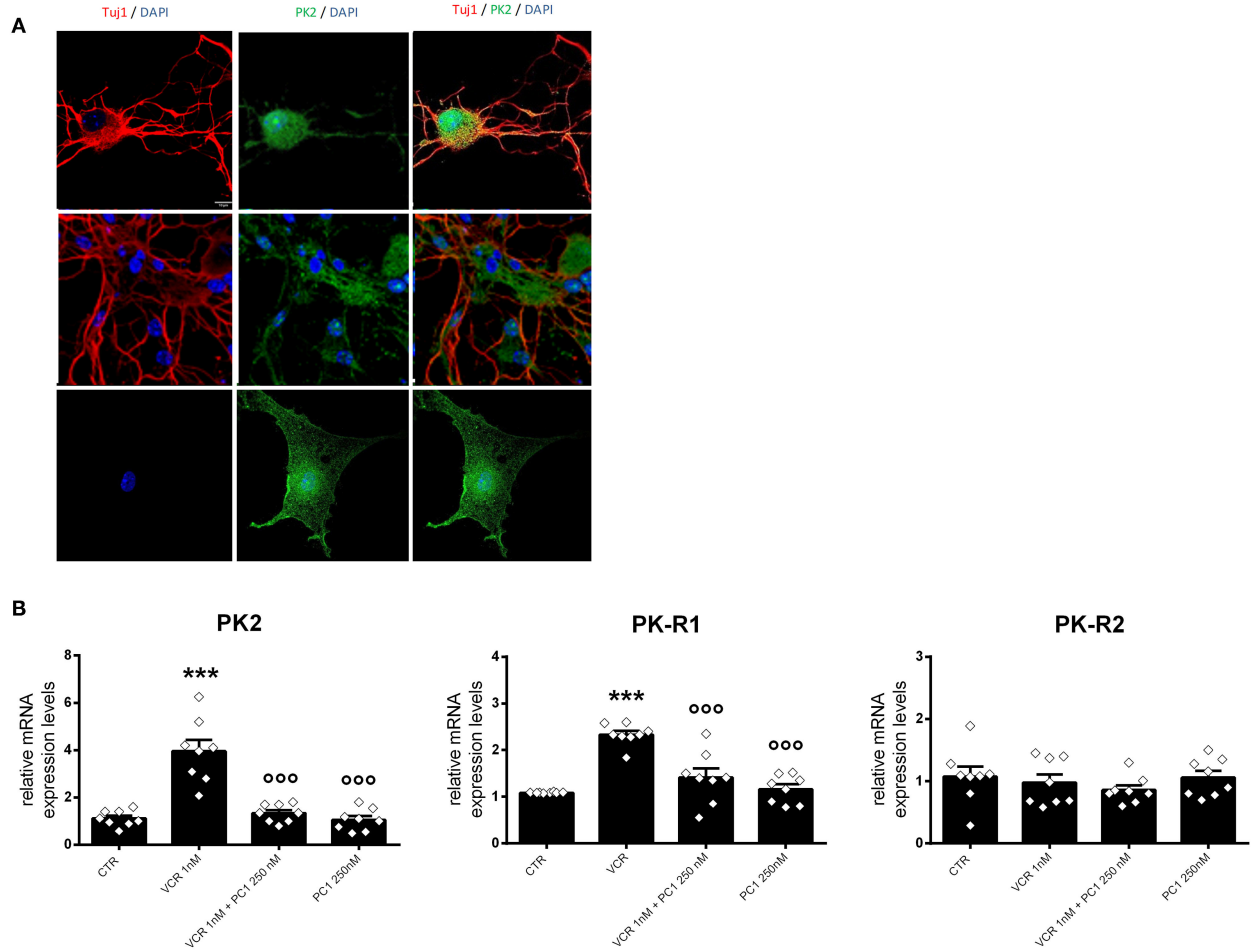
**(A)** Shows representative images of β3-tubulin (Tuj1; red), PK2 (green), and DAPI (blue) signal in vehicle-treated DRG cell cultures. Scale bar = 10 μm. **(B)** represents the expression levels of PK2, PK-R1, and PK-R2 that were measured in DRG cultures treated with vehicle (CTR), VCR 1 nM alone, or in combination with PC1 250 nM (VCR + PC1) and PC1 250 nM without the chemotherapeutic agent. mRNA levels, determined by RT-qPCR, were expressed in relation to GAPDH and presented as fold increases over the levels of CTR condition (relative mRNA expression levels). Data are presented as mean ± SEM of eight cultures obtained from eight animals. Statistical analysis was performed by means of one-way ANOVA followed by Bonferroni's post-test. ***p < 0.001 vs. CTR; ^°°°^*p* < 0.001 vs. VCR 1 nM.

Given the results obtained in the neurite length assay with VCR and considering our previous study on the contribution of PK2 in VCR-induced neuropathy *in vivo* (31), we investigated the role of PK2 and its receptors in the neuronal alterations induced by VCR. As shown in Figure 3, neurons and non-neuronal cells expressed PK2 in culture (panel A). Cultures exposed to VCR 1 nM (panel B) displayed significant upregulation of PK2 [*F*_3, 28_ = 27.92, *p* < 0.001;CTR vs. VCR, *p* < 0.001] and PK-R1 [*F*_3, 28_ = 22.94, *p* < 0.001; CTR vs. VCR, *p* < 0.001] mRNA expression, while no differences were detected in PK-R2. When cells were simultaneously incubated with the PK-R inhibitor (PC1 250 nM; VCR 1 nM), both PK2 and PK-R1 upregulation was not present anymore (PK2: VCR vs. VCR + PC1, *p* < 0.001; PK-R1: VCR vs. VCR + PC1, *p* < 0.001). PC1 alone at the dose of 250 nM did not induce any changes in the expression levels of the PK system components (Figure 3B). This suggests critical involvement of PK2 and PK-R1 in the molecular mechanism of VCR neurotoxicity with an autocrine loop.

### Increased Expression of Neuroimmune Markers by VCR

**Figure 4:**
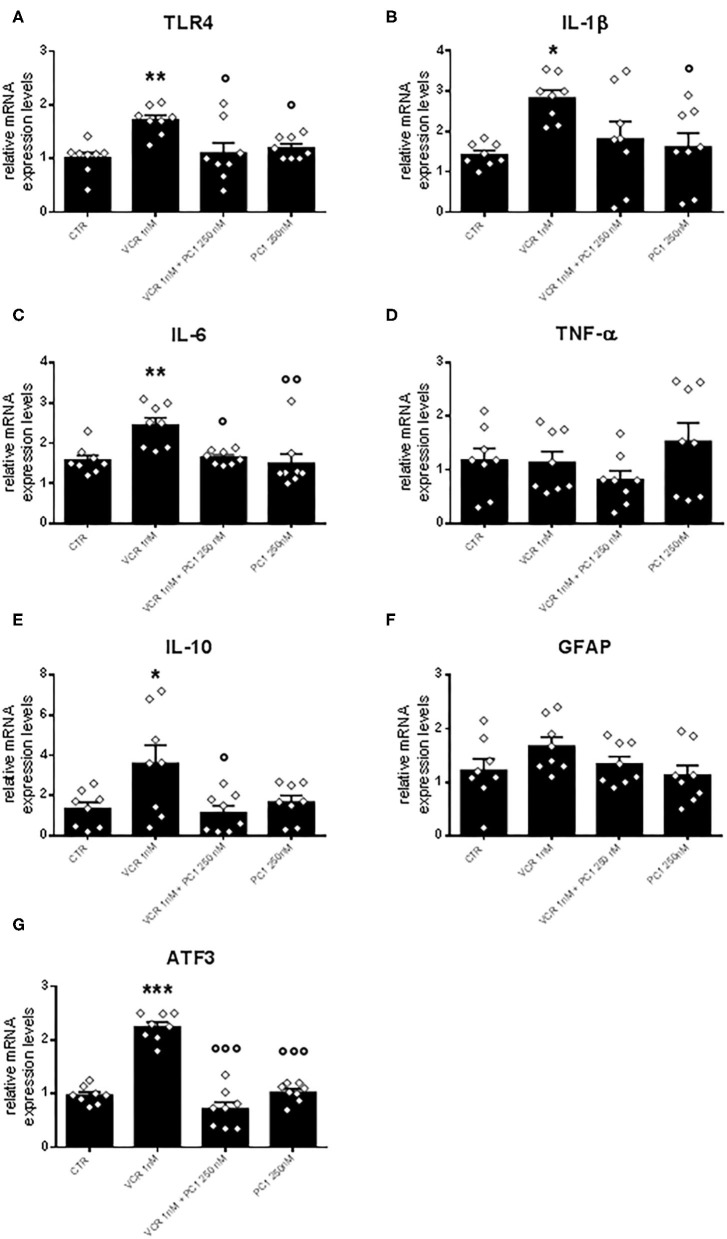
Expression levels of TLR4 **(A)**, IL-1β **(B)**, IL-6 **(C)**, TNF-α **(D)**, IL-10 **(E)**, GFAP **(F)**, and ATF3 **(G)**, measured in DRG cell cultures treated with vehicle, VCR 1 nM alone, or in association with PC1 250 and PC1 250 nM without the chemotherapeutic agent. mRNA levels, determined by RT-qPCR, were expressed in relation to GAPDH and presented as fold increases over the levels of CTR condition (relative mRNA expression levels). Data are the mean ± SEM of eight cultures obtained from eight mice. Statistical analysis was performed by means of one-way ANOVA followed by Bonferroni's post-test. *p < 0.05, **p < 0.01, ***p < 0.001 vs. CTR; °*p* < 0.05, ^°°^*p* < 0.01, ^°°°^*p* < 0.001 vs. VCR 1 nM.

In addition to PK2 and PK-R1, in the same cultures, VCR 1 nM induced upregulation of several inflammatory and damage markers such as TLR4 [*F*_3, 28_ = 6.355, *p* = 0.002; CTR vs. VCR, *p* = 0.0027], IL-1β [*F*_3, 28_ = 4.374, *p* = 0.012; CTR vs. VCR, *p* = 0.0153], IL-6 [*F*_3, 28_ = 7.195, *p* < 0.001; CTR vs. VCR *p* = 0.0051], IL-10 [*F*_3, 28_ = 4.383, *p* = 0.012; CTR vs. VCR, *p* = 0.0369], and ATF3 [*F*_3, 28_ = 60.800, *p* < 0.001; CTR vs. VCR, *p* < 0.001]. In this experimental condition (VCR 1 nM), no alterations in the expression of TNF-α or GFAP were observed (Figure 4). The changes induced by VCR (1 nM) were significantly reduced when cells were simultaneously incubated with 250 nM PC1 (VCR vs. VCR + PC1: TLR4, *p* = 0.010; IL-6, *p* = 0.011; IL-10, *p* = 0.019; ATF3, *p* < 0.001). These findings indicate that the PK system not only is involved in the autocrine regulation of PK2 and PK-R1 but also has a critical role in a downstream neuroinflammatory component of VCR neurotoxicity.

### Prevention of VCR-Induced Neurite Outgrowth Inhibition by PK-R Antagonist PC1

**Figure 5:**
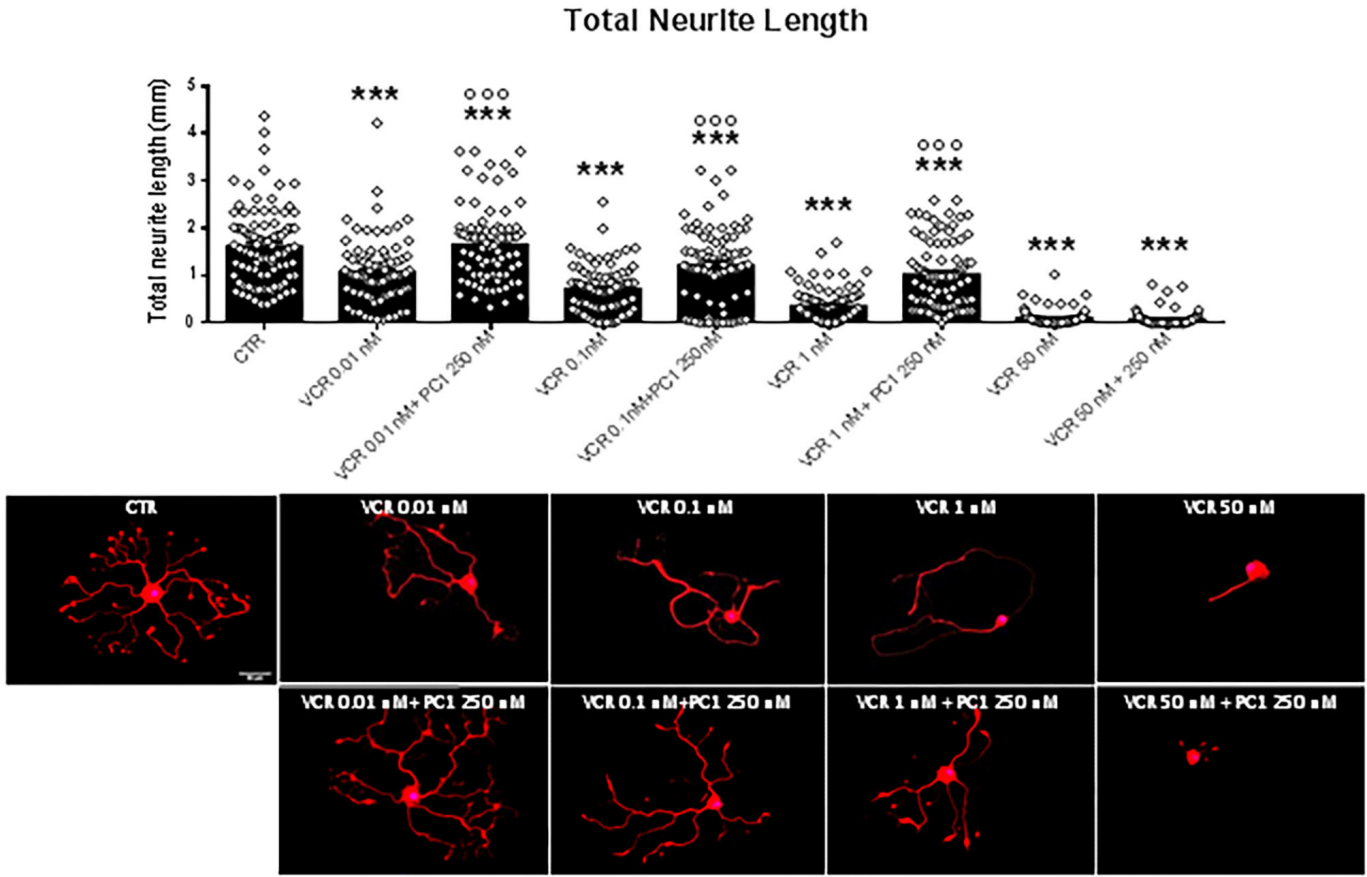
Quantification of total neurite length of DRG primary sensory neurons, when the PK-Rs antagonist was *in vitro* administered in combination with different VCR nanomolar concentrations (0.01, 0.1, 1, and 50 nM). In the same figure are reported representative images of β3-tubulin (Tuj1; red) staining in DRG cell cultures. Cell nuclei were counterstained with DAPI (blue). Scale bar = 50 μm. Mean ± SEM, *n* ≥ 80 cells obtained from four independent experiments. One-way ANOVA followed by Bonferroni's post-test. ***p < 0.001 vs. CTR; ^°°°^*p* < 0.001 vs. VCR relative dose.

Based on these findings, we tested PC1 together with VCR concentrations that induced a reduction of neurite outgrowth when compared not only to vehicle-treated cells but also one vs. the other (0.01, 0.1, 1, and 50 nM). PC1 was effective in reducing the neurite alterations induced by VCR 0.01, 0.1, and 1 nM [*F*_(8, 723)_ = 76.89, *p* < 0.001; VCR 0.01, 0.1, and 1 nM vs. VCR + PC1, *p* < 0.001], while no positive effect of PC1 250 nM was observed in neurons exposed to 50 nM VCR (Figure 5). This suggests that PK2 activation of PK-R1, as a consequence of VCR application, is a major component of the molecular mechanism involved in VCR neurotoxicity affecting sensory neurons.

### PC1 Prevents Neurotoxic BTZ Effects on Sensory Neuron Fibers

**Figure 6:**
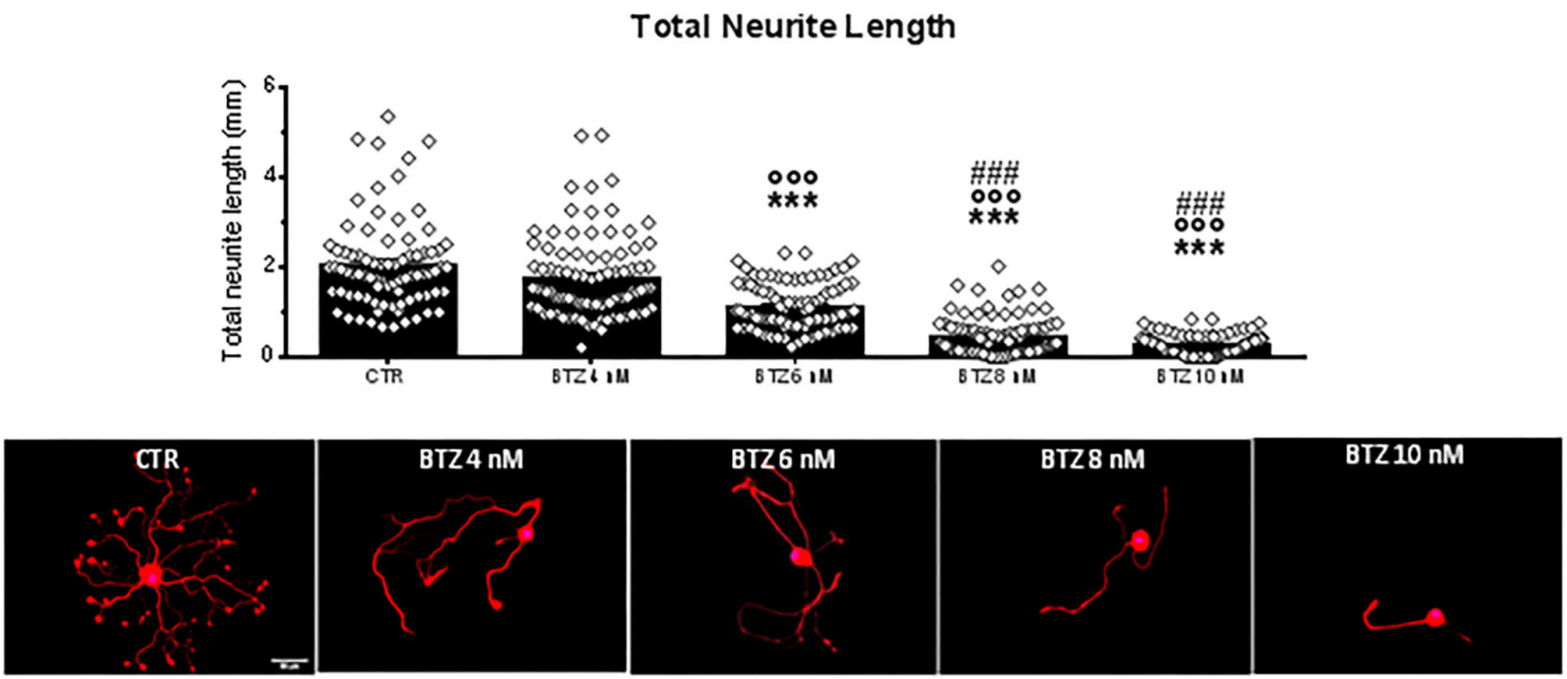
Quantification of total neurite length of DRG primary sensory neurons exposed *in vitro* to the vehicle (CTR) and 4 nanomolar doses of BTZ. In the same figure are reported representative pictures of β3-tubulin (Tuj1; red) staining in DRG cell cultures treated *in vitro* with the vehicle (CTR) or with the following BTZ concentrations: 4, 6, 8, and 10 nM. Cell nuclei were counterstained with DAPI (blue). Scale bar = 50 μm. Data are presented as mean ± SEM, *n* ≥ 80 cells obtained from four independent experiments. One-way ANOVA followed by Bonferroni's post-test. ***p < 0.001 vs. CTR; ^°°°^*p* < 0.001 vs. BTZ 4 nM; *p* < 0.001 vs. BTZ 6 nM.

**Figure 7:**
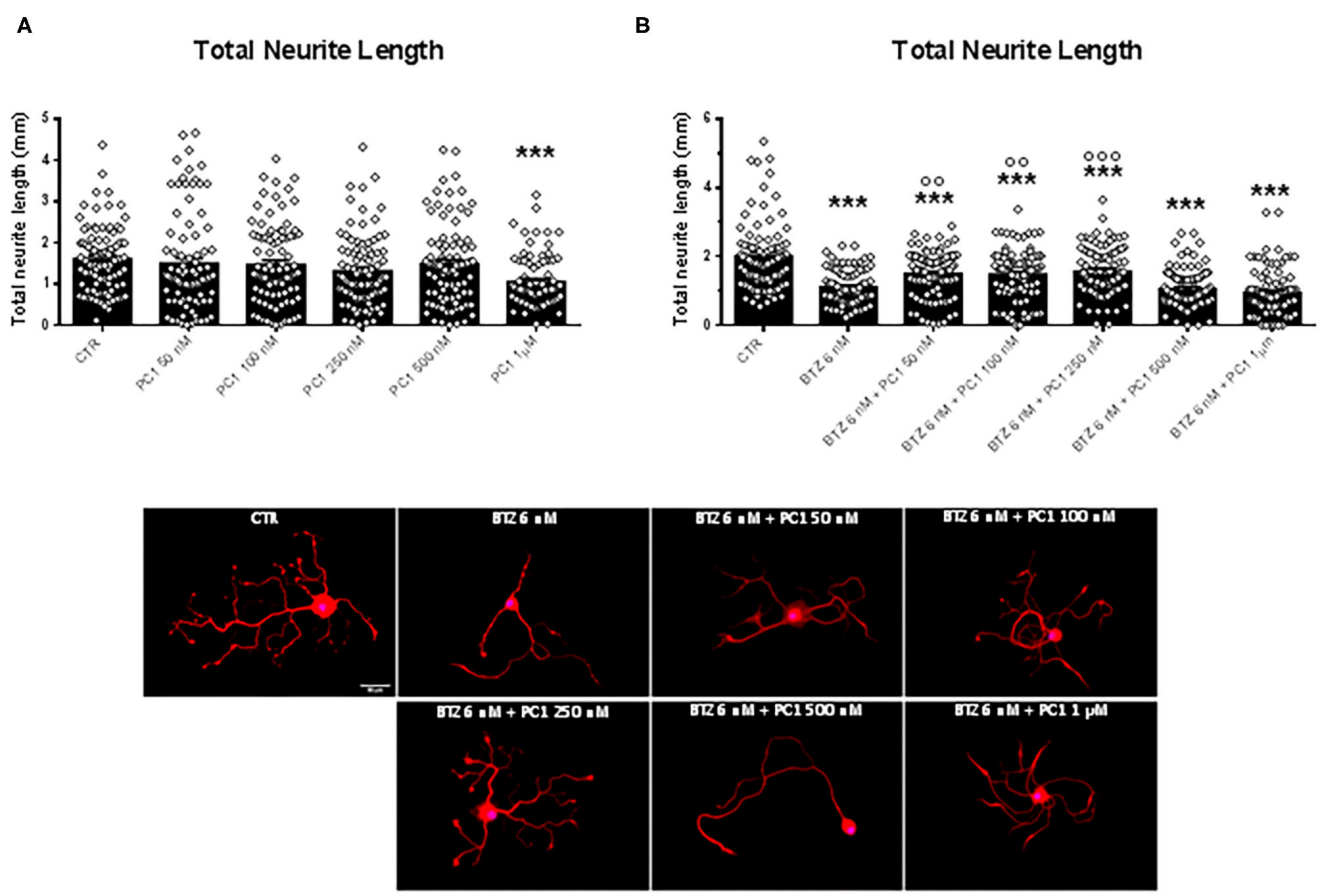
Quantification of total neurite length of CTR cells and the effect of different doses (50 nM, 100 nM, 250 nM, 500 nM, and 1 μM) of the PK-Rs antagonist PC1 on DRG primary sensory neurons outgrowth, *in vitro* administered alone **(A)** or in combination with BTZ 6 nM **(B)**. In the same figure are reported representative images of β3-tubulin (Tuj1; red) staining in DRG cell cultures exposed to the vehicle (CTR), BTZ 6 nM, and BTZ 6 nM in combination with PC1 50 nM, PC1 100 nM, PC1 250 nM, PC1 500 nM, and PC1 1 μM. Cell nuclei were counterstained with DAPI (blue). Scale bar = 50 μm. Data are presented as mean ± SEM, *n* ≥ 80 cells obtained from four independent experiments. One-way ANOVA followed by Bonferroni's post-test. ***p < 0.001 vs. CTR; ^°°^*p* < 0.01, ^°°°^*p* < 0.001 vs. BTZ 6 nM.

In order to validate the neuroprotective effects of PC1, we used BTZ as an antineoplastic drug with different mechanism of action but also able to induce CIPN. We assessed the neurite outgrowth in DRG primary sensory neuron cultures treated with four different nanomolar doses (4, 6, 8, and 10 nM) of BTZ (Figure 6). Cells exposed for 72 h to the lowest BTZ concentration (4 nM) did not show any decline in total neurite length in comparison to CTR. However, by increasing the concentration of the chemotherapeutic drug (BTZ 6 nM), a significant decrease of total neurite length was detected [*F*_(4, 437)_ = 114.80, *p* < 0.001; CTR vs. BTZ 6 nM, *p* < 0.001], and with higher BTZ concentrations (8 and 10 nM), the neurotoxic effect on neurite length was further aggravated (CTR vs. BTZ 8 and 10 nM, *p* < 0.001; BTZ 6 nM vs. BTZ 8 nM and 10 nM *p* < 0.001). Since BTZ 6 nM induced about 50% reduction in neurite length, this concentration was used to investigate the protective effects of PC1. Rising concentrations of PC1 (50, 100, 250, 500 nM, and 1 μM) were tested alone (Figure 7A) or co-applied with BTZ 6 nM for 72 h (Figure 7B). PC1 alone did not modify neurite outgrowth up to 500 nM, and only at the highest dose did PC1 itself slightly reduce neurite length (panel A). However, PC1 significantly counteracted the BTZ-induced reduction of total neurite length (panel B). At lower PC1 doses (50 and 100 nM), a slight but significant increase of total neurite length compared to BTZ-only treated cells was already evident [*F*_(6, 623)_ = 23.04, *p* < 0.001; BTZ vs. BTZ + PC1 50 nM, *p* = 0.0048 and vs. BTZ + PC1 100 nM, *p* = 0.0089]. Increasing the concentration of the antagonist to 250 nM, a stronger improvement was observed (BTZ vs. BTZ + PC1 250 nM, *p* = 0.0002).

## Discussion

In the present study, we found that the PK-R antagonist PC1 was able to prevent the neurotoxic effect exerted on primary sensory neurons by two different chemotherapeutic drugs, VCR and BTZ in *vitro*. An upregulation of PK2 and PK-R1 but not PK-R2 receptors was present in VCR-treated cultures, and the PK-R antagonism significantly reduced this upregulation of PK2 and PK-R1 receptor along with an additional effect on upregulated neuroimmune mediators. PK2 localizes in both neurons and non-neuronal cells, possibly satellite cells, present in the culture. This is in accordance with literature showing the presence of PK2 and its receptors (PK-R1 and PK-R2) in both neurons and surrounding accessory cells (25, 34, 47, 53). In addition, a functional association between PK2, TRPV1, and TRPA1 in DRG neurons has been reported (29, 53, 54). PK2 and PK-Rs in DRG have been found to be expressed mostly, although not uniquely, in small peptidergic neurons (29). From our immunofluorescence study, PK2 positive staining appears to be present in all cultured neurons, although further experiments better assessing localization of PK2 in different types of neurons, such as peptidergic or not peptidergic neurons, would be of interest. Our findings are in line with our recent *in vivo* studies on CIPN induced by BTZ (30) and VCR (31) in mice, demonstrating not only that the PK system is involved in the maintenance of CIPN induced by these two drugs but also that inhibition of PK-Rs ameliorates the signatures of pain behavior and reduces the neuroimmune activation at the level of the peripheral and central nervous system. PK2 can be upregulated by different pathological factors, such as hypoxia, reactive oxygen species, and excitotoxic glutamate, leading to the hypothesis that PK2 could have a general role as an insult-inducible endangering mediator for neuronal damages (32–35). It is now well accepted that the free nerve endings of primary sensory afferents, but also satellite cells, are susceptible to anticancer agents, such as BTZ and VCR, because of the lack of the blood–brain barrier and the presence of fenestrated capillaries, which lead to an unrestrained drug permeation (3, 13–15). For this reason, in addition to *in vivo* animal models (55), numerous *in vitro* studies have been carried out, mainly on sensory neuron cultures, in order to investigate the molecular mechanisms at the basis of chemotherapy-induced neurotoxicity (36, 52). Here, we used the adult DRG neuron *in vitro* model and the quantification of total neurite length as morphological read-out that correlates with neuron function and health (56). In general, a small percentage of non-neuronal cells, such as satellite cells and macrophages are contaminating in this type of culture (38–40). In order to obtain pure neuronal cultures, the use of an anti-mitotic agent is needed, but this is considered a toxic treatment that can interfere with neuronal viability (40, 57–60, 71). Furthermore, the presence of satellite cells in DRG cultures is useful because it resembles more closely the *in vivo* environment of sensory neurons (36, 39, 61). Cytostatic drugs turn down tumor growth via different molecular mechanisms targeting tumor cell division and survival. Despite the different mechanisms of action, the majority of these drugs are neurotoxic. Both VCR and BTZ induce neurotoxic damage, as demonstrated by the increased levels of cell stress as indicated by upregulation of the damage marker ATF3 found by us and others (5, 30, 31, 62). Chemotherapeutic agents can directly bind to toll like receptor 4 (TLR4) (13, 63), which triggers the activation of several pro-inflammatory pathways. The upregulation of pro-inflammatory cytokines, such as IL-6 and IL-1β, subsequently induces the activation of STAT3 that binds to the PK2 promoter and induces the activation and further production of PK2 (64). Since reactive oxygen species (ROS) can induce PK2 expression (32) and VCR has been constantly shown to activate ROS in neurons (4), we can also hypothesize that VCR-induced ROS may contribute to PK2 overexpression. The present study for the first time presents evidence that this pathway can be relevant for the damage induced in sensory neurons since increased levels of PK2 were identified in VCR-treated neuron cultures and the increase was abolished by PC1. Once activated, PK2 is able to sustain its own production in a positive loop and to amplify the pro-inflammatory signal as demonstrated by several *in vivo* and *in vitro* studies (22, 26, 65, 66). In line with these reports, PC1 reduced not only expression of PK2 and PK-R1 but also that of several pro-inflammatory cytokines, suggesting that PK2 via PK-R1 may act as upstream regulator of cytokine expression. Based on our results, we propose that the PK-R antagonist PC1, by selectively binding to PK2 receptors, reduces or even prevents the propagation of a “damage signal” induced by chemotherapeutics. Apparently, in the presence of PC1, the cell stress and injury response characterized by an upregulation of pro-inflammatory cytokines and ATF3 was profoundly reduced. The specificity of the antagonist and the lack of off-target effects are supported by several published evidences. *In vivo*, when administered alone in normal mice, PC1 did not induce behavioral/biochemical alterations (18, 22). The antagonist did not counteract hypersensitivity induced by PGE2, ATP, or bradykinin. Other PK-Rs peptidic or non-peptidic antagonsists exert anti-inflammatory and modulatory effects similar to those induced by PC1 *in vivo* and *in vitro* (21, 47, 67, 68). All these results seem to indicate that PC1 is effective in blocking PK2 activity when endogenously overexpressed.

We are aware that a limitation of this study could be that the PK system, markers, and cytokines are evaluated only as mRNA levels. However, in our previous works that evaluated PK system and pro-inflammatory mediators in DRG in *ex vivo* experiments, mRNA and protein were similarly modulated (18, 21, 30). Moreover, other authors, using primary cortical neurons, demonstrated a parallel modulation of the PK system as mRNA and protein (34, 47). Despite their different anti-neoplastic mechanisms of action, both chemotherapeutic agents used in our study induced a dose-dependent decline of neurite length. However, VCR had a prominently stronger neurotoxic effect than BTZ. This is in accordance with clinical data in which VCR appears to be more toxic and induces CIPN in a larger number of patients at lower doses compared to BTZ (69, 70).

Interestingly, our results confirm the high vulnerability of DRG to the neurotoxic effect of chemotherapeutics and suggest that DRG-resident cells, both neuronal and non-neuronal, are sufficient to trigger and sustain a neuroinflammatory condition. In addition, also the protective effect on axonopathy obtained by PK blocking is exerted without the involvement or recruiting of infiltrating peripheral immune cells.

In conclusion, our findings suggest that the PK system contributes to the neurotoxic effect started in sensory neurons by anti-neoplastic agents underlying chemotherapy-induced neuropathic pain and sensory deficits. Blocking PK2 signaling by PC1 emerges as a promising therapeutic approach as this molecule not only reduces signatures of CIPN *in vivo* (30, 31) but also, by protecting neurons, could prevent and suppress the causes underlying progression of neuropathic pain during chemotherapeutic treatment.

## Data Availability Statement

All datasets generated for this study are included in the article/supplementary material.

## Ethics Statement

The animal study was reviewed and approved by Animal Care and Use Committee of the Italian Ministry of Health (permission number 709/ 2016) and the Austrian National Animal Experiment Ethics Committee of the Austrian Bundesministerium für Wissenschaft und Forschung (permit number BMWF-66.011/0054-WF/V/3b/2015).

## Author Contributions

GM and TK: methodology, data curation, and formal analysis. GM wrote the original paper. GA: data curation, review, and editing. RL: review. PS and MK: conceptualization, supervision, and writing—review and editing. SF: supervision, funding acquisition, and writing—review and editing. All authors contributed to the article and approved the submitted version.

## Conflict of Interest

The authors declare that the research was conducted in the absence of any commercial or financial relationships that could be construed as a potential conflict of interest.
